# Transcriptional factor OCT4 promotes esophageal cancer metastasis by inducing epithelial-mesenchymal transition through VEGF-C/VEGFR-3 signaling pathway

**DOI:** 10.18632/oncotarget.18035

**Published:** 2017-05-20

**Authors:** Chunguang Li, Maoling Zhu, Xiaoli Lou, Chunying Liu, Hezhong Chen, Xuejing Lin, Weidan Ji, Zhigang Li, Changqing Su

**Affiliations:** ^1^ Department of Thoracic Surgery and Reconstructive Surgery, Changhai Hospital, Second Military Medical University, Shanghai 200433, China; ^2^ Department of Molecular Oncology, Eastern Hepatobiliary Surgery Hospital and National Center of Liver Cancer, Second Military Medical University, Shanghai 200433, China; ^3^ Department of Gastroenterology, Shanghai Yangpu Hospital, Tongji University, Shanghai 200090, China; ^4^ Department of Thoracic Surgery, Shanghai Chest Hospital Esophageal Disease Center, Shanghai Jiao-Tong University, Shanghai 200030, China

**Keywords:** esophageal carcinoma, epithelial-mesenchymal transition, octamer-binding transcription factor 4, metastasis, signaling

## Abstract

The octamer-binding transcription factor 4 (OCT4) can promote cancer proliferation and metastasis. Esophageal carcinoma (ECC) harbors different quantities of OCT4-positive cancer cells. These cells are highly malignant and prone to metastasis; however, the mechanism remains unknown. In this study, we found that OCT4 enhances vascular endothelial growth factor C (VEGF-C) promoter activity to promote VEGF-C expression and activates VEGF receptor 3 (VEGFR-3) in ECC cells, thereby inducing cancer cell epithelial-mesenchymal transition (EMT). Studies using xenograft models showed that OCT4 promoted xenograft growth and intraperitoneal implantation metastasis of ECC cells. Downregulation of OCT4 expression could inhibit cancer metastasis. OCT4- and VEGF-C-positive ECC presented more malignant biological behaviors and the corresponding patients exhibited a poor prognosis. The study confirmed that the OCT4/VEGF-C/VEGFR-3/EMT signaling plays a role in the progression of ECC. Understanding of how OCT4 regulates EMT and how ECC metastasis occurs will provide useful targets for the biological treatment of ECC.

## INTRODUCTION

Esophageal carcinoma (ECC) is a malignant gastrointestinal cancer with morbidity second only to gastric cancer. At present, surgery is the main treatment for ECC, but the five-year survival rate remains at 15% to 25%, and relapse and metastasis are the main causes of patient death [1]. Our preliminary studies indicated that a small population of octamer-binding transcription factor 4 (OCT4)-positive cancer cells was present in ECC tissues and that ECC with a high percentage of OCT4-positive cells displayed rapid progression, high incidence of lymph node metastasis, and short tumor-free survival and overall survival [2]. OCT4 is one of the key transcription factors of the Pit-Oct-Unc (POU) family. As a stem cell marker and an important factor for pluripotency maintenance, OCT4 plays an important role in regulating and maintaining self-renewal and multi-directional differentiation of embryonic stem cells [3]. Recent studies showed that OCT4 is expressed in human solid tumors, including lung cancer [4], breast cancer [5], and head and neck cancer [6], and especially in germ cell tumors, such as embryonal carcinoma and seminoma, and is closely related to tumor proliferation, metastasis and prognosis [7]. For ECC tissues, the OCT4-positive rate was approximately 26%, and OCT4 inhibited apoptosis of cancer cells by increasing survivin expression [8]. Moreover, OCT4 promoted cyclin D1 (CCND1) expression and activated cyclin-dependent kinase 4/6 (CDK4/6) activity to accelerate cell cycle progression and promote the proliferation and division of cancer cells [8]. OCT4 expression in solid tumors supported the theory of cancer stem cells (CSCs) and became a highly sensitive and highly specific marker and a treatment target for malignant tumors. OCT4 can be regarded as an oncogene with complex mechanisms in its role in tumor development and progression; besides its role in regulating survivin and CCND1-related signaling pathways (as our study showed), OCT4 may promote the malignant progression of tumors by regulating the Wnt, Hedgehog, and transforming growth factor (TGF)-β signaling pathways [9].

Lymphatic metastasis is most common and is also the main factor to affect long-term survival of ECC patients. Currently, it is known that ECC with high OCT4 expression is prone to lymph node metastasis, but the mechanism is not clear. Studies have shown that cancer cells with stem cell characteristics, especially the OCT4- and NANOG-positive cancer cells, may undergo epithelial-mesenchymal transition (EMT) and rapid metastasis [10, 11]. In recent years, the role of EMT in the metastasis of epithelium-derived malignant tumors has become a research hotspot. Studies on the molecular mechanisms of ECC development and progression have provided a growing body of evidence for the important role of EMT. EMT refers to the biological process in which epithelium-derived cells transform into cells of a mesenchymal phenotype; this process plays a critical role in embryonic stem cell differentiation, tissue repair and regeneration, organ fibrosis, and tumor development and progression. Cancer cells undergoing EMT present some changes in phenotype and biological behaviors, including low homogeneous adhesion, high invasion and migration, low or absent expression of characteristic epithelial molecules (such as E-cadherin and keratin), and significantly increasing expression of characteristic mesenchymal molecules (such as vimentin and N-cadherin) [12]. EMT confers invasion and migration activity and anoikis resistance for cancer cells, maintains the pluripotency of cancer cells and promotes the self-renewal of CSCs [13, 14]. Therefore, EMT is believed to be an early event of invasion and metastasis of epithelial tumors.

In this study, we investigated the molecular mechanism of ECC metastasis and found that OCT4 induced EMT in ECC cells and promoted cancer metastasis. Moreover, we established a new treatment strategy for curbing cancer metastasis by blocking the pathway of the upstream regulatory molecules involved in cancer metastasis. The results demonstrated that OCT4 activated the vascular endothelial growth factor (VEGF)-C/VEGF receptor 3 (VEGFR-3) signaling pathway, thus inducing EMT in ECC cells and promoting the lymphatic metastasis of cancer cells.

## RESULTS

### OCT4 expression level was closely related to the invasion activity of ECC cells

**Figure 1:**
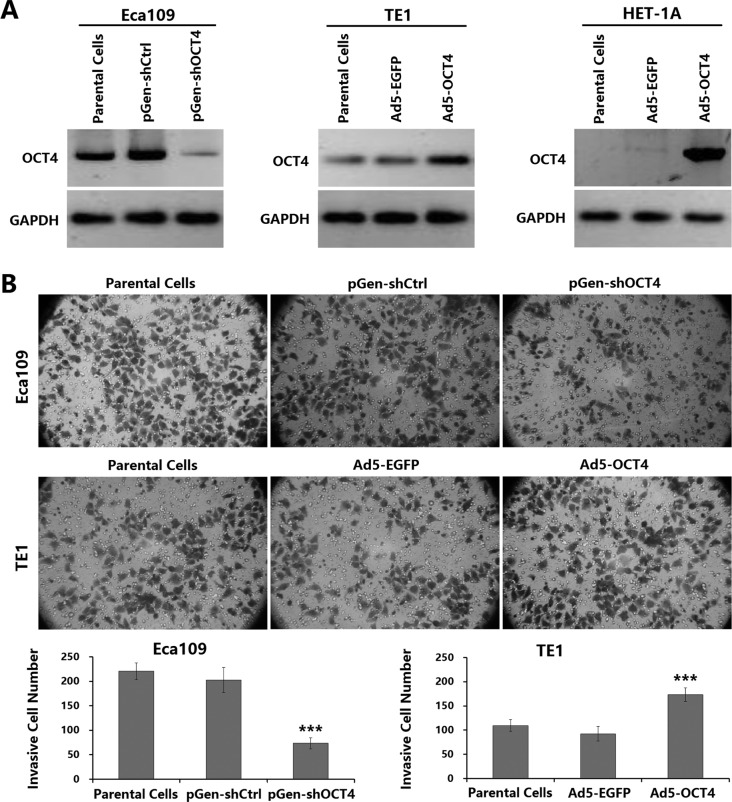
OCT4 overexpression enhanced the invasion activity of ECC cells (**A**) Human ECC cell lines (Eca109 and TE1) and normal esophageal cell line (HET-1A) were seeded in 24-well plates at a density of 1 × 10^5^ cells in each well, cultured for 24 h, and transfected with shRNA vectors at a concentration of 20 μg/well or infected with adenoviruses at a multiplicity of infection (MOI) of 100 pfu/cell as indicated. After cultured for 48 h, cells were harvested and total protein was extracted. The expression level of OCT4 was detected by western blotting. Glyceraldehyde-3-phosphate dehydrogenase (GAPDH) was used as a loading control. (**B**) ECC cell invasive ability was measured by Transwell assay. The ECC parental cells and the shRNA vector-transfected or adenovirus-infected sub-cell lines were seeded in upper chamber of Transwell at a density of 5 × 10^4^ cells/200 μl per chamber. The lower chamber was added with 500 μl medium. After 48 h of culture, the invasive cells were stained with 0.1% crystal violet for 15 min, and five high-power fields (objective lens 20×) were randomly selected under a microscope to count cell number; ***p < 0.001 *versus* the parental cell group.

To verify the relationship between the OCT4 expression level and the invasion ability of ECC cells, we performed a Transwell experiment to detect cell invasion ability. Both Eca109 cells and TE1 cells were OCT4 positive, but OCT4 expression was significantly stronger in Eca109 cells than in TE1 cells; HET-1A cells did not express OCT4 (Figure 1A). The invasion ability was stronger in Eca109 cells than in TE1 cells. The Eca109-shOCT4 cells which were transfected with pGen-shOCT4 to knock down OCT4 expression had a significantly decreased invasion ability compared with Eca109 parental cells. The TE1-OCT4 cells which were infected with Ad5-OCT4 to enhance OCT4 expression had a significantly increased invasion ability compared with TE1 parental cells (Figure 1B). These results demonstrated that the level of OCT4 expression was closely related to the invasion activity of ECC cells.

### OCT4 promoted the EMT of ECC cells

**Figure 2:**
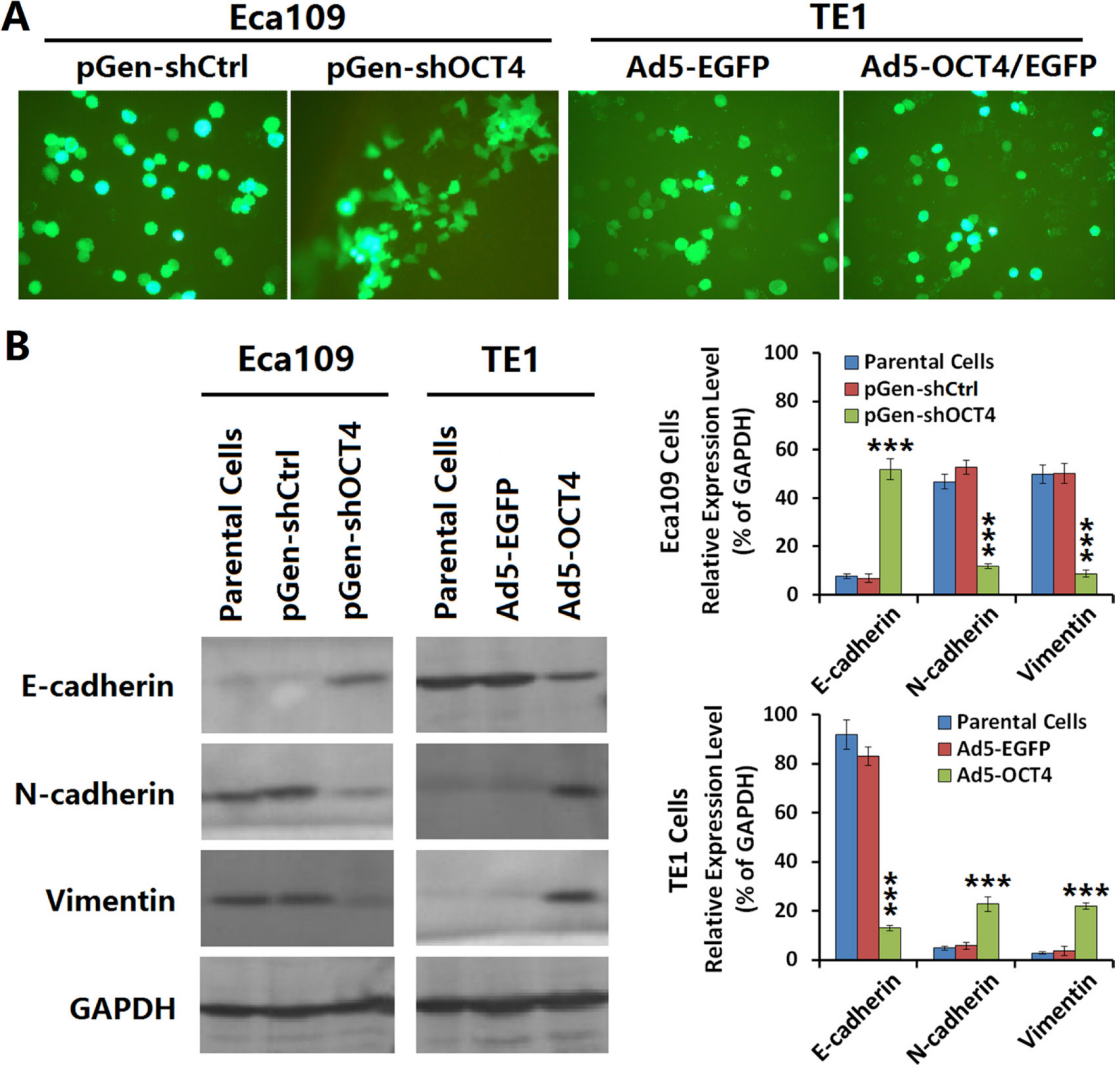
OCT4 induced the epithelial-mesenchymal transition (EMT) in ECC cells (**A**) The Eca109 and TE1 cells were seeded in 24-well plates at a density of 1 × 10^5^ cells in each well and cultured for 24 h. Eca109 cells were transfected with the indicated shRNA vectors at a concentration of 20 μg/well, and TE1 cells were infected with the indicated adenoviruses at an MOI of 100 pfu/cell. After cultured for 48 h, cells were observed under a fluorescent microscope; primary magnification: 200×. (**B**) Eca109 cells transfected with shRNA vectors and TE1 cells infected with adenoviruses as aforementioned were cultured for 48 h, the harvested cells were prepared to detect the expression levels of EMT markers by western blotting. GAPDH was used as a loading control and the relative expression levels of the indicated EMT markers were normalized to GAPDH; ***p < 0.001 *versus* the parental cell group.

Both pGen-shOCT4 and pGen-shCtrl vectors possessed an EGFP reporter gene, and we observed phenotypic changes in Eca109-shOCT4 cells which appeared to be more clustered adherent cells with intercellular pseudopodium contact (Figure 2A, left panel). The TE1 cells had not obviously changes in phenotype after infected with Ad5-EGFP and Ad5-OCT4 (Figure 2A, right panel). To elucidate the mechanism of these phenotypic changes in Eca109 cells, we compared the expression of EMT-related parameters between Eca109 cells and Eca109-shOCT4 cells and between TE1 cells and TE1-OCT4 cells. The results showed that E-cadherin expression was lower in Eca109 cells than in TE1 cells and that N-cadherin and vimentin expression was lower in TE1 cells than in Eca109 cells. Moreover, E-cadherin expression level was significantly increased in Eca109-shOCT4 cells compared with Eca109 cells, and N-cadherin and vimentin expression was significantly decreased in Eca109-shOCT4 cells compared with Eca109 cells. E-cadherin expression level was decreased and N-cadherin and vimentin expression was increased in TE1-OCT4 cells compared with TE1 cells (Figure 2B).

### OCT4 expression was related to the activity of VEGF-C/VEGFR-3 signaling pathway

**Figure 3:**
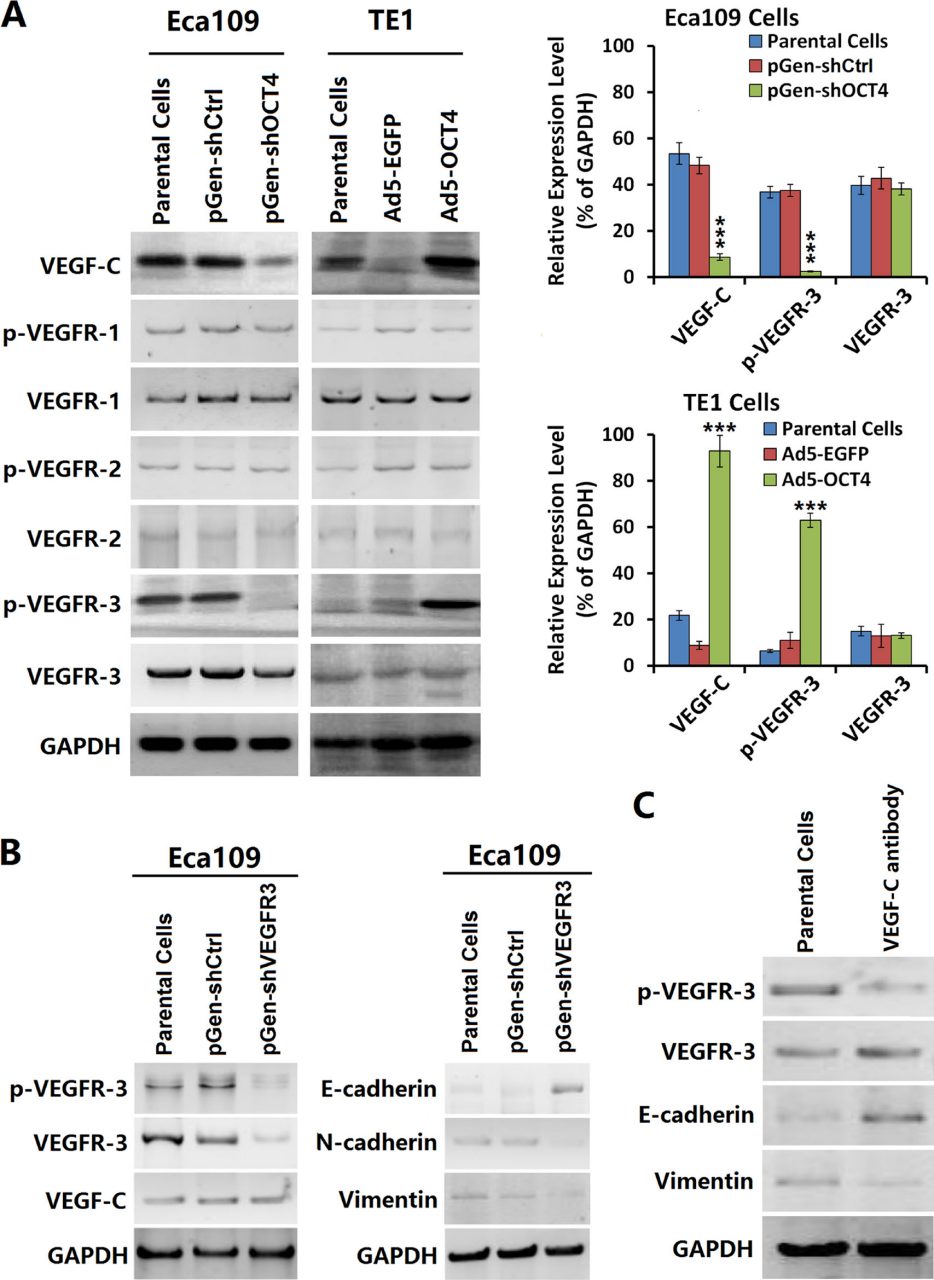
OCT4 increased VEGF-C expression and activated the VEGFR-3 signaling pathway (**A**) Eca109 and TE1 cells were cultured, and transfected with shRNA vectors or infected with adenoviruses as aforementioned in Figure 1A. After cultured for 48 h, the harvested cells were prepared to detect the contents of VEGF-C and a group of VEGFR members by western blotting. The relative expression level of every protein was normalized to GAPDH; ***p < 0.001 *versus* the parental cell group. (**B**) Eca09 cells were cultured in 24-well plates at a density of 1 × 10^5^ cells in each well for 24 h, then transfected with pGen-shVEGFR3 and pGen-shCtrl at a concentration of 20 μg/well. After cultured for 48 h, cells were harvested for detecting the expression levels of the indicated proteins by western blotting. GAPDH was used as a loading control. (**C**) Eca109 cells were incubated with the VEGF-C antibody at a concentration of 100 μg/well in 24-well plates and cultured for 48 h. Cells were harvested and detected the expression levels of indicated factors by western blotting. GAPDH was used as a loading control.

After OCT4 expression was knocked down with a shRNA vector, we observed a decreased level of VEGF-C and p-VEGFR-3 in Eca109-shOCT4 cells but no significant change in p-VEGFR-1 or p-VEGFR-2 levels. In contrast, after OCT4 expression was enhanced with an adenoviral vector, VEGF-C and p-VEGFR-3 levels were increased in TE1-OCT4 cells (Figure 3A). Moreover, the p-VEGFR-3 level was higher in Eca109 parental cells. After VEGFR-3 and p-VEGFR-3 levels were down-regulated with a VEGFR-3-specific shRNA vector, we observed no significant change in VEGF-C expression but a significantly increased level of E-cadherin expression and decreased levels of N-cadherin and vimentin expression (Figure 3B). We incubated the Eca109 cells with the VEGF-C neutralization antibody and found that p-VEGFR-3 was reduced (Figure 3C). These results suggested that VEGF-C regulated the EMT process of cancer cells via VEGFR-3 phosphorylation.

### OCT4 promoted VEGF-C expression by regulating the VEGF-C promoter activity

**Figure 4:**
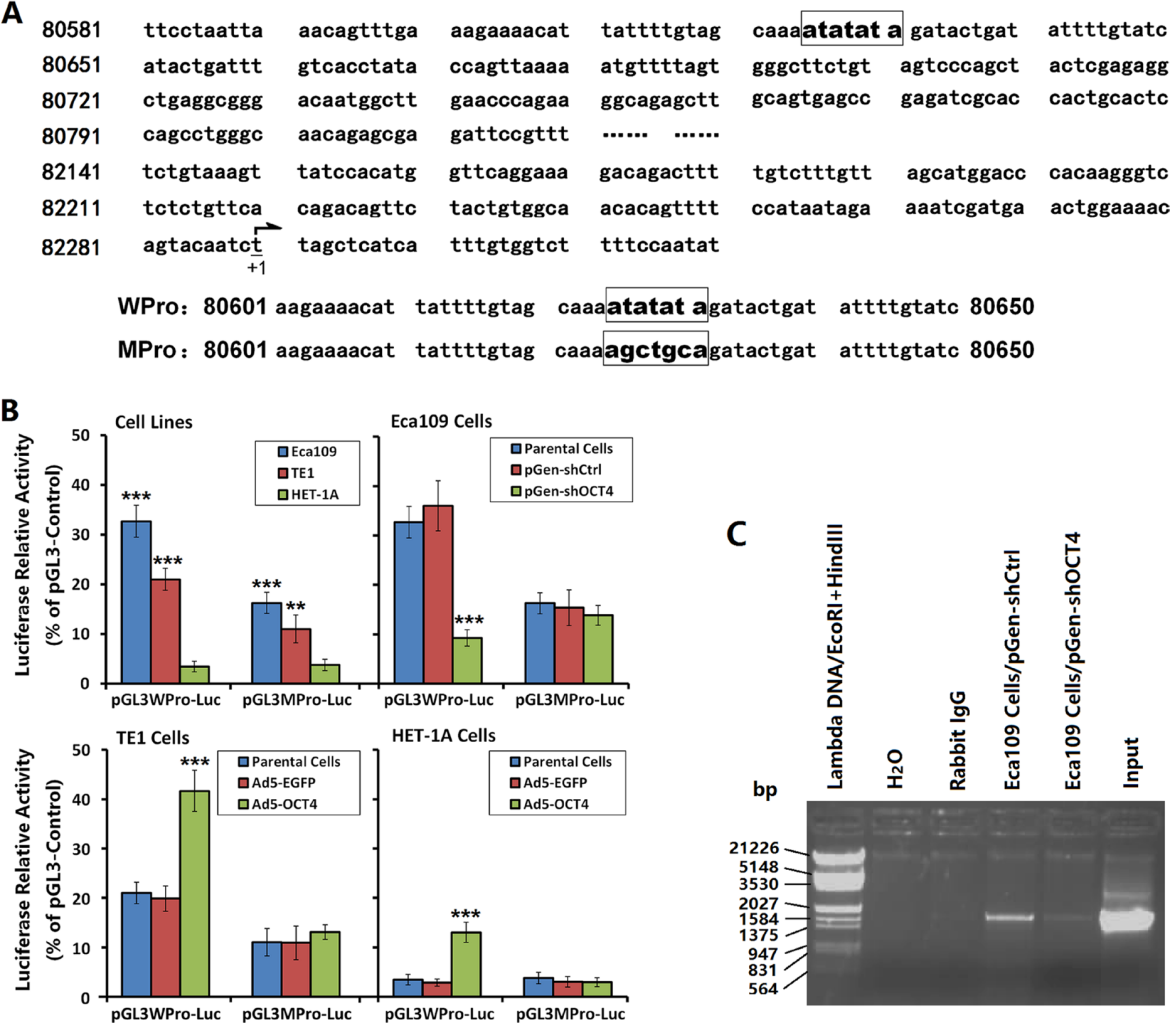
OCT4 promoted VEGF-C expression by regulating the VEGF-C promoter activity (**A**) Luciferase vectors contained the wild-type VEGF-C promoter (pGL3WPro-Luc) and the mutant promoter (pGL3MPro-Luc) were constructed. The nucleotides 80625-80631 (ATATATA) in pGL3WPro-Luc were mutated to “AGCTGCA” in pGL3MPro-Luc. “+1” indicated the transcription start site. (**B**) Eca109, TE1 and HET-1A cells were cultured and transfected with shRNA vectors or infected with adenoviruses as aforementioned in Figure 1A. After cultured for 48 h, cells were transfected with pGL3WPro-Luc or pGL3MPro-Luc at 200 ng/well and co-transfected with pRL-TK at 20 ng/well. After a continuous culture for 48 h, the harvested cells were performed to detect the luciferase activity by the Dual-Luciferase Reporter Assay System. The plasmid pGL3-Control was used as a positive control and the relative luciferase activity of the WPro and MPro promoters was normalized to pGL3-Control; **p < 0.01 and ***p < 0.001 *versus* the HET-1A cells in left-most image and *versus* the parental cells in right 3 images. (**C)** ChIP assay in Eca109 cells was performed to show the binding of OCT4 to the VEGF-C promoter. Immunoprecipitation of chromatin DNA fragments was carried out with anti-OCT4 antibody. The input chromatin sample was used as a positive control. The rabbit IgG instead of anti-OCT4 antibody and the distilled water instead of immunoprecipitated chromatin DNA sample were used as negative controls.

Gene sequence analysis showed no octamer motif but, rather, TA repeats as “ATATATA” in the VEGF-C promoter region (Figure 4A). We constructed luciferase fluorescent reporter vectors regulated by the WPro or MPro promoters to determine whether OCT4 controlled the activity of VEGF-C promoter in ECC cells. The results showed that the WPro promoter activity was significantly higher in Eca109 cells and TE1 cells than in HET-1A cells, and was higher in Eca109 cells than in TE1 cells. In contrast, the MPro promoter activity was significantly decreased in Eca109 cells and TE1 cells compared with the WPro promoter activity, with no significant change in HET-1A cells. After OCT4 knockdown in Eca109 cells, the WPro promoter activity was significantly decreased, with no significant change in the MPro promoter activity. After OCT4 overexpression in TE1 cells and HET-1 A cells, the WPro promoter activity was increased, with no significant change in the MPro promoter activity (Figure 4B).

ChIP assay was performed to further identify the binding of OCT4 to the VEGF-C promoter. The results showed that the anti-OCT4 antibody immunoprecipitated DNA sample from the pGen-shCtrl-transfected Eca109 cells could be amplified a corresponding band as that of amplification with input positive control, but no band was amplified in the negative controls. After knockdown of OCT expression, the amplified band in the anti-OCT4 antibody immunoprecipitated DNA sample was decreased markedly (Figure 4C).

### OCT4 promoted ECC xenograft growth and metastasis in nude mice

**Figure 5:**
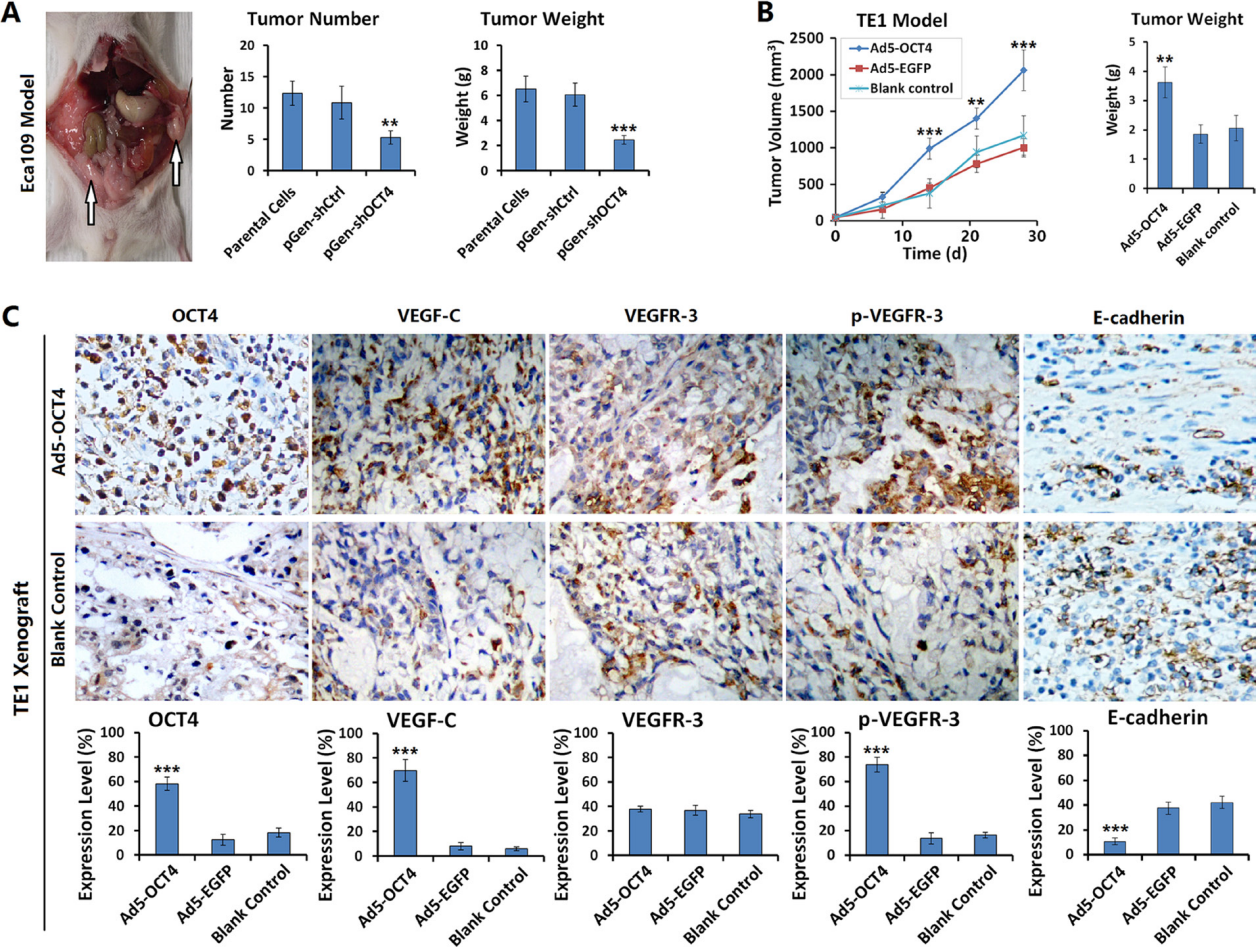
OCT4 promoted ECC xenograft growth and metastasis in nude mice (**A**) The Eca109 parental cells, pGen-shOCT4-transfected and pGen-shCtrl-transfected Eca109 cells were intraperitoneally injected into nude mice at 1 × 10^6^ cells/100 μl/mouse, with 5 mice in each group. Three weeks later, mice were sacrificed and dissected abdominal cavity to count and weigh the xenograft tumors; **p < 0.01 and ***p < 0.001 *versus* the parental cells. (**B**) TE1 cells were subcutaneously injected into the right armpits of nude mice at 1 × 10^6^ cells/100 μl/mouse. After the xenograft tumors formed at day 10, mice were randomly divided into 3 groups (Ad5-OCT4, Ad5-EGFP, blank control), with 5 mice in each group. The first two groups were intratumorally injected with the corresponding adenovirus, at 1 × 10^8^ pfu/100 μl, once a day for 10 days, the control group was injected with saline synchronously at the same volume. Tumor size was measured weekly and tumor volume was calculated with a formula “maximum diameter × minimum diameter^2^ × 0.5”. At day 28 after first treatment, the experiment was terminated and tumor specimens were removed and weighed; **p < 0.01 and ***p < 0.001 *versus* the blank control group. (**C**) The TE1 xenograft tumors were fixed in 10% formalin, paraffin-embedded, and sectioned for detecting the expression levels of the indicated proteins by immunohistochemistry. All of the sections were observed within five high-power fields (objective lens 20×) to count the positive cells; primary magnification: 200×; ***p < 0.001 *versus* the blank control group.

We established ECC xenograft models in nude mice to observe xenograft growth, metastasis, and changes in the activity of signaling pathways after the intervention of OCT4 expression. The results showed that the number and weight of intraperitoneal implantation metastatic tumors were significantly decreased in Eca109-shOCT4 cells than in Eca109 parental cells (Figure 5A). With OCT4 overexpression, the TE1 xenografts grew faster. At 14 days after Ad5-OCT4 injection, tumor volume was significantly higher in the treatment group than in the control group. By day 28, tumor volume in the Ad5-OCT4 group of TE1 model exceeded the specification; therefore, the experiment was terminated, and the tumors were removed and weighed. The results demonstrated that tumors weighed significantly more in the Ad5-OCT4 group than in the control group (Figure 5B). TE1 xenograft tumors were sectioned to determine the expression levels of OCT4, VEGF-C, VEGFR-3, p-VEGFR-3 and E-cadherin with immunohistochemical staining. The results showed that Ad5-OCT4-mediated OCT4 overexpression significantly increased the positive levels of VEGF-C and p-VEGFR-3, and decreased the expression level of and E-cadherin, with no significant change in VEGFR-3 expression (Figure 5C), Supplementary Figures 1 and 2

### OCT4 overexpression promoted the lymphatic metastasis of ECC and affected prognosis

**Figure 6:**
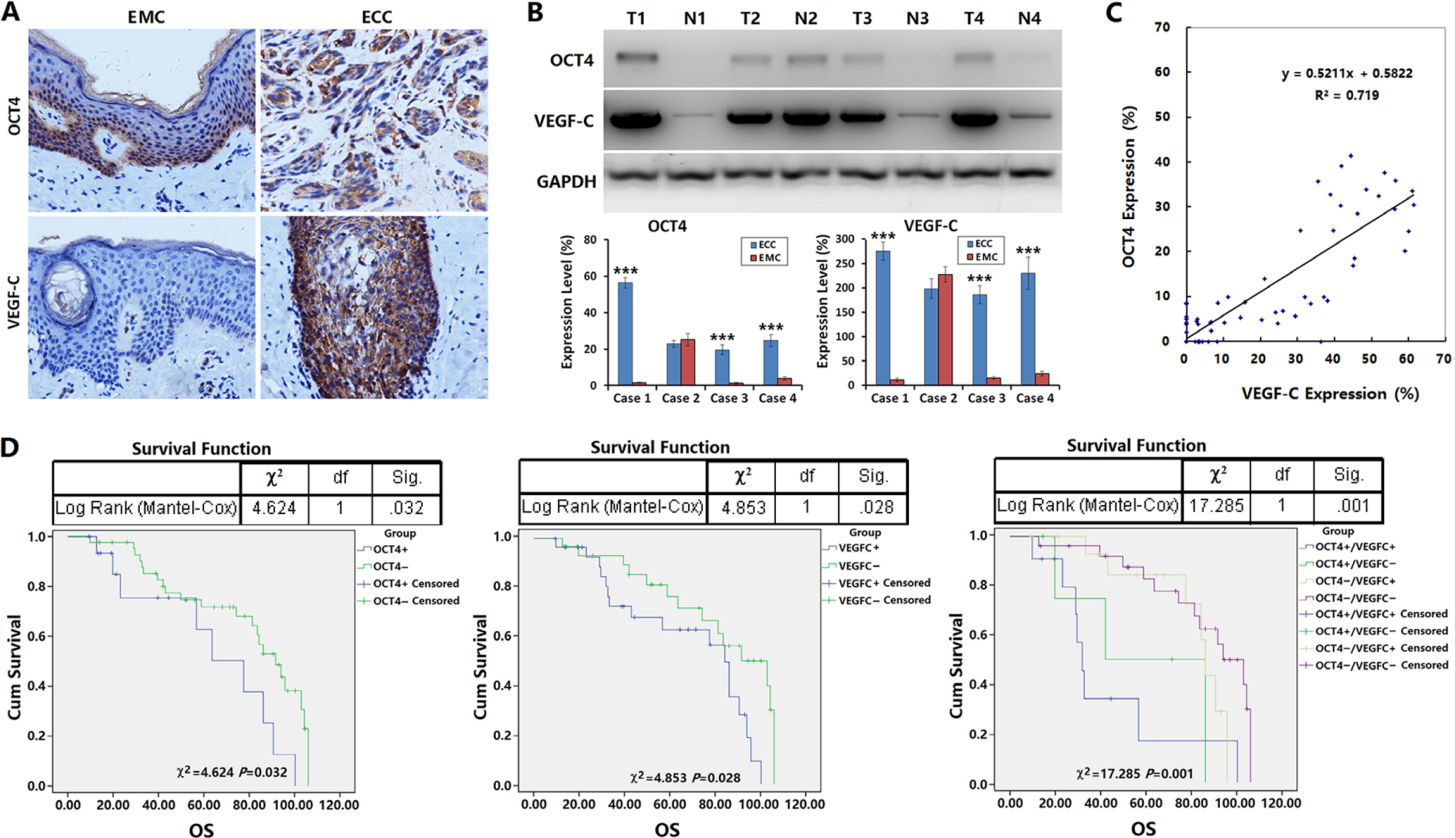
OCT4 promoted the lymphatic metastasis of ECC and affected prognosis of patients (**A**) The expression levels of OCT4 and VEGF-C were detected in 67 cases of surgical ECC specimens and paracancer esophageal mucosa (EMC) by immunohistochemistry; primary magnification: 200×. (**B**) Ten g of every ECC tissue and paracancer esophageal mucosa (EMC) was used to isolate total protein for examining the expression levels of OCT4 and VEGF-C by western blotting. The relative expression levels of OCT4 and VEGF-C were normalized to GAPDH. T: ECC tissue, N: EMC tissue; ***p < 0.001 *versus* the corresponding EMC; Error bar: standard deviation from three independent experiments. (**C**) The percentages of positive cells for OCT4 and VEGF-C in ECC tissues by immunohistochemistry were counted to analyze the linear correlation between OCT4 and VEGF-C expression. (**D**) The relationships between the expression of OCT4, VEGF-C and the overall survival of ECC patients were illustrated through Kaplan-Meier survival curves and analyzed by a log-rank test.

To further investigate the regulation of OCT4 on the activity of VEGF-C/VEGFR-3 signaling pathway and the effect on the biological behaviors of ECC cells, we determined the expression levels of OCT4 and VEGF-C with immunohistochemistry in the primary tumor tissues from 67 cases of surgical ECC specimens. The results showed that the OCT4-positive rate was 28.36% (19/67) in ECC tissues and only 5.97% (4/67) in matched esophageal mucosal tissues (EMC), the OCT4-positive rate was significantly higher in cancer tissues than in paracancer tissues (*p* = 0.0006). The OCT4-positive reaction was mainly concentrated in the ECC cell nuclei and cytoplasm. For esophageal mucosal epithelia, OCT4-positive cells were located at the base (Figure 6A, upper panel), Supplementary Figure 3. The VEGF-C-positive rate was 50.75% (34/67) in ECC tissues and significantly higher than that in EMC tissues (50.75% *versus* 10.45%, *p <* 0.0001; Figure 6A, lower panel). Western blotting confirmed that OCT4 and VEGF-C levels were significantly higher in ECC tissues than in EMC tissues (Figure 6B).

We analyzed the relationship between OCT4 or VEGF-C expression level and the clinicopathological characteristics of ECC. The results showed that the level of OCT4 expression was related to the level of ECC differentiation, lymph node metastasis and clinical stage. OCT4 expression was observed in higher levels in the patients with poor differentiation of cancer cells, positive lymph node metastasis and late clinical stage. The level of VEGF-C expression was related to lymph node metastasis of ECC and clinical stage, with higher level in the patients with lymph node metastasis and late clinical stage. Although VEGF-C expression was unrelated to the differentiation of cancer cells, the VEGF-C expression level showed an increasing trend in the poorly differentiated ECC. The expression of OCT4 and VEGF-C was unrelated to the patients’ gender, age and distant metastasis (Table 1). Further analysis showed that the expression of OCT4 and VEGF-C was positively correlated; that is, VEGF-C expression was significantly higher in OCT4-positive ECC tissues (Figure 6C).

**Table 1:** Correlation between clinicopathological characteristics and expression of OCT4 and VEGF-C in ECC

Clinicopathological characteristics	*n*	OCT4		*p* Value	VEGF-C		*p* Value
		Positive (%)	Negative		Positive (%)	Negative	
Gender							
Male	52	15 (28.85)	37	0.8690	25 (48.08)	27	0.4158
Female	15	4 (26.67)	11		9 (60.00)	6	
Age							
≥ 64*	34	8 (23.53)	26	0.3734	16 (47.06)	18	0.5400
< 64	33	11 (33.33)	22		18 (54.55)	15	
Differentiation							
Well	23	3 (13.04)	20	0.0058^#^	8 (34.78)	15	0.1360
Intermediate	29	7 (24.14)	22		17 (58.62)	10	
Poorly	15	9 (60.00)	6		9 (60.00)	8	
Lymph node metastasis							
No metastasis	28	4 (14.29)	24	0.0304^#^	10 (57.98)	18	0.0370^#^
Metastasis	39	15(38.46)	24		24 (46.32)	15	
Distant metastasis							
No metastasis	55	14 (25.45)	41	0.2589	25 (45.45)	30	0.0636
Metastasis	12	5 (41.67)	7		9 (75.00)	3	
Clinical stage							
Early	18	2 (11.11)	16	0.0030^#^	5 (27.78)	13	0.0336^#^
Intermediate	37	9 (24.32)	28		20 (54.05)	17	
Late	12	8 (66.67)	4		9 (75.00)	3	

Notes: *, median age; ^#^, chi-square test.

In this study, 59 of the 67 ECC patients had clinical follow-up data. The follow-up period was 9 to 106 months, and the median overall survival was 65 months. During follow-up, 54 patients relapsed or progressed, and 32 patients died. Kaplan-Meier analysis showed that the levels of OCT4 and VEGF-C were related to overall survival (OCT4: *p* = 0.032; VEGF-C: *p* = 0.028), and the positive OCT4 and VEGF-C expression indicated poorer prognosis in ECC patients (*p* = 0.001; Figure 6D), Supplementary Tables 1–3.

## DISCUSSION

According to the CSC theory, cancer tissues harbor a few group of cells with unlimited proliferation, self-renewal and multi-potential differentiation, which is the source of tumor relapse and metastasis [15, 16]. CSCs of various tumors can commonly express certain cell surface antigenic markers (such as CD133, CD90, CD44, ABCG2, etc.) or transcription factors (such as Oct-3/4, Nanog, Sox2, nestin, c-myc, c-kit, β-catenin, etc.), but the expression levels or amount of these parameters vary greatly among different tumor tissues. OCT4 is expressed in a variety of tumors including breast cancer, bladder cancer, prostate cancer, liver cancer, head and neck squamous cell cancer, non-small cell lung cancers, and ECC [15, 17]. OCT4 is a transcription factor of the POU family. It promotes tumor cell proliferation and inhibits cell apoptosis; additionally, OCT4 induces tumor invasion and metastasis. As a DNA-binding protein, OCT4 recognizes the cis-regulatory elements, that is the octamer motif (ATGCAAAT) or the TA-rich sequence, in the promoter or enhancer of target genes to regulate target gene transcription and then to activate or inhibit the activity of various signaling pathways, such as TGF-β1, Wnt/β-catenin/Snail, RTK/Ras/MAPK, Notch, Hedgehog, and PI3K/AKT/mTOR, finally promoting tumor development and progression [9, 18–20]. Our preliminary studies showed that there is a few OCT4-positive cancer cells in ECC tissues, thus providing possible direct evidence of CSC existence [2]. ECC cases that harbor a large amount of OCT4-positive cells progress rapidly and are highly invasive and prone to relapse and metastasis with a poor prognosis. Recently, the *in vitro* cytological studies showed that OCT4 directly regulates the biological behaviors, such as proliferation, invasion, and metastasis, of a variety of tumors. Our study on ECC demonstrated that OCT4 promoted survivin expression to inhibit apoptosis in cancer cells [2] and promoted CCND1 expression and activated CDK4/6 activity to accelerate cell cycle progression [8]. However, it is not yet clear how OCT4 promotes ECC metastasis, especially lymph node metastasis.

The VEGF family comprises a group of growth factors that specifically act on vascular endothelial cells. They are secreted glycoproteins, including VEGF (also known as VEGF-A), VEGF-B, VEGF-C, VEGF-D, VEGF-E, placental growth factor (PlGF), and platelet-derived growth factor (PDGF), in which VEGF-C stimulates lymphatic endothelial cell proliferation and induces lymphangiogenesis [21]. Studies have shown that VEGF-C is related to lymphatic metastasis of a variety of malignant tumors. For malignant tumors, such as breast cancer, gastric cancer, prostate cancer, pancreatic cancer, cervical cancer, non-small cell lung cancer, and throat cancer, high VEGF-C expression promotes lymphatic invasion and lymph node metastasis of cancer cells [22–24]. Moreover, high VEGF-C expression down-regulates the expression of epithelial phenotypic markers of cancer cells and up-regulates the expression of mesenchymal phenotypic markers, thus presumably promoting cancer development by inducing EMT in cancer cells [25]. VEGF-C promotes lymphangiogenesis in the surrounding area of cancer nests, whereas cancer cells undergoing EMT are often located at tumor edges, providing further evidence of the correlation between VEGF-C and EMT [26]. Studies have demonstrated high VEGF-C expression in ECC tissues, but further research is needed to investigate the regulatory mechanism and the effect of VEGF-C on the biological behaviors of cancer cells.

For cancer cells, EMT is a complex process regulated by multi-level signaling molecules. Studies using ECC cell lines have found varying amounts of OCT4-positive cells in different ECC cell lines. OCT4 activates the VEGF-C promoter activity to promote VEGF-C expression, which, in turn, acts as a ligand to activate VEGFR-3 phosphokinase activity, thus inducing EMT in cancer cells. We noted that OCT4 overexpression enhanced ECC invasion and metastasis ability, whereas OCT4 knockdown in ECC cells significantly inhibited VEGF-C expression and reduced p-VEGFR-3 level in cancer cells, thereby inhibiting tumor metastasis. ECC nude mouse xenograft experiments also showed that OCT4 overexpression promoted tumor growth and that OCT4 knockdown inhibited intraperitoneal implantation metastasis of cancer cells. Therefore, the relationship between OCT4 and EMT, as well as the regulatory role of OCT4 on ECC metastasis, offers new targets for biological cancer therapy. To further verify the effect of OCT4 on the biological behaviors of ECC, we studied clinical specimens obtained from 67 ECC patients to analyze the relationship between OCT4 or VEGF-C expression and the patients’ clinicopathological characteristics. The results showed that the OCT4-positive rate was significantly higher in ECC patients with poorly differentiated cancer cells, lymph node metastasis, or late clinical stage; the VEGF-C positive rate was significantly higher in cancer tissues with lymph node metastasis than that without lymph node metastasis. Follow-up data showed that the expression of OCT4 and VEGF-C was related to patient prognosis, as the overall survival was significantly shorter in patients with positive OCT4 and VEGF-C expression. These results confirmed that during ECC development and progression, OCT4 is an important transcription factor involved in the EMT process that can enhance ECC invasion and metastasis by inducing EMT and maintaining pluripotency of cancer cells.

Taken together, these data suggest that the OCT4/VEGF-C/VEGFR-3/EMT signaling pathway plays a regulatory role in ECC development and progression. OCT4 promotes VEGF-C expression and activates the VEGFR-3 phosphokinase activity and the downstream signaling pathways, thus inducing EMT in cancer cells and ultimately enhancing tumor invasion and metastasis, promoting tumor progression, and severely affecting patient prognosis. We elucidate how OCT4 regulates EMT and how metastasis of ECC cells occur, thus providing a useful target for research on mechanism of ECC metastasis and on biological therapy of ECC patients.

## MATERIALS AND METHODS

### Cell lines and vectors

Human ECC cell lines (Eca109 and TE1) and normal esophageal cell line (HET-1A) were purchased from the Cell Bank of Chinese Academy of Sciences (Shanghai, China). Cells were maintained as a monolayer in RPMI-1640 with 10% fetal bovine serum (FBS), 100 IU/ml penicillin G and 100 mg/ml streptomycin, at 37°C in a humidified 5% CO_2_ incubator.

An experimental adenovirus carrying the full-length cDNA of OCT4 (Ad5-OCT4) and the corresponding control adenovirus Ad5-enhanced green fluorescent protein (Ad5-EGFP) were constructed previously [8]. The OCT4-shRNA plasmid (pGen-shOCT4) and the VEGFR-3-shRNA plasmid (pGen-shVEGFR3) were provided by Wuhan Genesil Biotechnology Co., Ltd. (Wuhan, China). The 19-nt sense DNA of OCT4-shRNA (5′-CCC TCA CTT CAC TGC ACT G-3′) targets the base pairs 1233–1253 of the OCT4 gene (GenBank: DQ486513.1) and the 19-nt sense DNA of VEGFR-3-shRNA (5′-GGA TGG AAA GGC ACT GTC C-3′) targets the base pairs 1140–1158 of the VEGFR-3 gene (GenBank: U43143.1). The mock control shRNA vector pGen-shCtrl (shCtrl: 5′-GAC TTC ATA AGG CGC ATG C-3′) was concomitantly constructed.

### Luciferase assay

The wild-type VEGF-C promoter (WPro; nucleotides 80581-82320, GenBank NC_000004) and the mutant promoter (MPro: nucleotides 80625-80631 “ATATATA” in WPro were mutated to “AGCTGCA”) were synthesized and inserted into a luciferase reporter gene vector pGL3-Basic (Promega Co., Madison, WI, USA) to construct pGL3WPro-Luc and pGL3MPro-Luc.

ECC cells were seeded in 24-well plates at a density of 1 × 10^5^ cells in each well and cultured for 24 h. The Eca109 cells were transfected with pGen-shOCT4 and pGen-shCtrl at a final concentration of 20 μg/well. The TE1 cells were infected with adenoviruses Ad5-OCT4 and Ad5-EGFP at a viral multiplicity of infection (MOI) of 100 pfu/cell. After an additional 48 h culture, cells were transfected with pGL3WPro-Luc and pGL3MPro-Luc (200 ng/well) and co-transfected with pRL-TK (20 ng/well). The positive control plasmid pGL3-Control (Promega Co.) was used as a positive control. The cells were continuously cultured for 48 h and the harvested cells were performed according to the kit instructions of the Dual-Luciferase Reporter Assay System (Promega Co.) to determine the luciferase activity.

### Western blotting

ECC cells were cultured and transfected with shRNA vectors or infected with adenoviruses as aforementioned. The harvested cells were used to extract total cellular protein according to the manufacturer's instructions of the protein extraction reagent kit (Pierce Biotechnology, Inc., Rockford, IL, USA). The expression levels of proteins were detected by western blotting using the primary antibodies, including the mouse anti-OCT4 (Santa Cruz Biotechnology, Inc., Santa Cruz, CA, USA); the mouse anti-E-cadherin, mouse anti-N-cadherin, mouse anti-Vimentin, rabbit anti-VEGF-C (Cell Signaling Technology, Danvers, MA, USA); the mouse anti-VEGFR-1, mouse anti-VEGFR-2, mouse anti-VEGFR-3, rabbit anti-phospho-VEGFR-1, rabbit anti-phospho-VEGFR-2, rabbit anti-phospho-VEGFR-3 (Cell Applications Inc., CA, USA).

### Chromatin immunoprecipitation (ChIP) assay

The shRNA vector-transfected Eca109 cells were seeded in 6-well plates at 1 × 10^6^ cells/well and subjected to prepare the chromatin samples after incubated with 1% formaldehyde for 10 min at room temperature. The chromatin samples were immunoprecipitated with anti-OCT4 antibody. The negative control rabbit IgG (Cell Signaling Technology, Inc.) and distilled water were used as negative controls, and the soluble chromatin prior to immunoprecipitation was used as an input positive control. The immunoprecipitated DNA samples were analyzed by PCR using the primers (forward: 5′-ctaattaaacagtttg-3′; reverse: 5′-gtccatgctaacaa ag-3′). The product was 1,616 bp in length.

### Immunohistochemistry

Paraffin-embedded sections of ECC clinical specimens and nude mouse xenograft tumors were used to determine the expression levels of OCT4, VEGF-C, VEGFR-3, and phospho-VEGFR-3 (p-VEGFR-3) with a streptavidin-peroxidase immunohistochemical assay; the same antibodies were used as those in western blotting. All of the sections were observed within five high-power fields (objective lens 20×) to count and calculate the percentage of positive cells, any case with the positive cells less than 10% was defined as a negative case.

### Cell invasion assay

A Transwell (8 μm pore size, Corning, Tewksbury, USA) was placed in 24-well plates. The upper chamber of Transwell was covered with 50 μl of 1:6 diluted matrigel (BD Biosciences, San Jose, USA), then the ECC parental cell lines and the adenovirus-infected or shRNA vector-transfected sub-cell lines were seeded at a density of 5 × 10^4^ cells/200 μl per chamber. The lower chamber was added with 500 μl medium containing 10% FBS. The upper chamber was removed after 48 h of culture and stained with 0.1% crystal violet for 15 min, and five high-power fields (objective lens 20×) were randomly selected under a microscope to count cell number. The experiment was repeated 3 times.

### ECC xenograft experiment in nude mice

The animal experiments were strictly conducted in accordance with the Guide for the Care and Use of Laboratory Animals of the Second Military Medical University, and were approved by Committee of Laboratory Animal Center of the Second Military Medical University (SMMU20150127). Thirty-five healthy, male, 4-week-old purebred BALB/C nude mice were provided by the Shanghai SLAC Experimental Animal Center, Chinese Academy of Sciences. For Eca109 model, the Eca109 parental cells, pGen-shOCT4-transfected Eca109 cells and pGen-shCtrl-transfected Eca109 cells were intraperitoneally injected into nude mice at 1 × 10^6^ cells/100 μl/mouse, with 5 mice in each group. Three weeks later, the mice were anesthetized, sacrificed and dissected abdominal cavity to observe the formation of tumor implantation metastasis. The number of tumor nodules was counted and the total weight of all tumors was weighed. For TE1 model, the TE1 cell suspension was subcutaneously injected into the right armpits of 20 nude mice at 1 × 10^6^ cells/100 μl/mouse. The xenografts formed in all of the mice at day 10 after cell implantation, with an average diameter of 0.48 ± 0.09 cm. Three mice with the largest tumors and two mice with the smallest tumors were excluded, and the remaining 15 mice were randomly divided into 3 groups (Ad5-OCT4 group, Ad5-EGFP group, blank control group). The Ad5-OCT4 and Ad5-EGFP groups received multi-point intratumor injections with the corresponding adenovirus, at 1 × 10^8^ pfu/100 μl, once a day for 10 days; the control group was injected with saline synchronously at the same volume. After treatment, tumor size was measured weekly, and tumor volume was calculated with a formula “maximum diameter × minimum diameter^2^ × 0.5”. The experiment would be terminated immediately if the mean tumor volume of any group exceeded the threshold (2,000 mm^3^) specified by the Ethics Committee for Experimental Animals of the Second Military Medical University. At the end of observation period, the mice were anesthetized and sacrificed, and tumor specimens were removed and weighed. Tumor tissues were fixed in 10% neutral formalin, paraffin-embedded, and sectioned for immunohistochemical examination.

### Patients and specimens

A total of 67 ECC patients who underwent surgery at Changhai Hospital between January 2005 and December 2009 were included in this study. The patients did not receive preoperative chemotherapy or radiotherapy. There were 52 male patients and 15 female patients (male to female: 3.47:1), aged 45 to 73 years old (median: 64 years old). Histopathological examination confirmed squamous cell carcinoma in all 67 cases. According to the TNM staging criteria of malignant tumors developed by the American Joint Committee on Cancer (AJCC) and the International Union Against Cancer (UICC), 2 patients were in stage 0 (TisN0M0), 6 in stage I (T1N0M0), 18 in stage II (10 in IIA: T2N0M0; 8 in IIB: T1-2N1M0), 29 in stage III (T3N1M0, T4N0-1M0), and 12 in stage IV (IVA: T1-4N0-1M1a; IVB: T1-4N0-1M1b). Stage 0, I and IIA were defined as early stage, stage IIB and III as intermediate stage, and stage IV as late stage. According to the level of cell differentiation, 23 cases were well-differentiated, 29 were intermediately-differentiated, and 15 were poorly-differentiated. According to metastasis, lymph node metastasis was present in 39 patients and absent in 28 patients, distant metastasis was present in 12 patients and absent in 55 patients. Patients with lymph node metastasis, distant metastasis, or other postoperative relapses underwent radiation therapy and/or chemotherapy. Fifty-nine patients had follow-up records from the time of diagnosis through December 31, 2015, with a median follow-up time of 81 months (9–106 moths). Overall survival (OS) was defined as from the time of diagnosis to the end of follow-up, or to death, or to the last follow-up date for patients who were lost-to-follow-up.

For surgical specimens, cancer tissues and paracancer tissues (3 cm or more away from cancer tissues) were collected. A portion of fresh tissues was used to extract protein, which was stored at −80°C, for western blotting; another portion of tissues was fixed in formalin, paraffin-embedded, and serially sectioned for HE staining and immunohistochemistry.

### Statistical analysis

The experimental data were collected from 3 times of independent *in vitro* experiments, as well as the *in vivo* experimental data from 5 mice per group. The data were analyzed by one-way analysis of variance (ANOVA). When the data were statistically different among the multiple groups, the SNK-q test was used to conduct the multiple comparison. The clinicopathological parameters were evaluated by chi-square test, and the patients’ OS was calculated by Kaplan-Meier method and compared through the log-rank test. The statistical analysis software package PASW Statistics 18 was used. The *p* values less than 0.05 were considered statistically significant.

## SUPPLEMENTARY FIGURES AND TABLES


