# Supplementary material for: Transcriptional factor OCT4 promotes esophageal cancer metastasis by inducing epithelial-mesenchymal transition through VEGF-C/VEGFR-3 signaling pathway
Source: Oncotarget. 2017 May 20;8(42):71933–45. doi: 10.18632/oncotarget.18035 (PMC5641101; doi:10.18632/oncotarget.18035)
Supplement: Supplementary file 1 [file oncotarget-08-71933-s001.pdf]

Transcriptional factor OCT4 promotes esophageal cancer metastasis by inducing epithelial-mesenchymal transition through VEGF-C/VEGFR-3 signaling pathway

SUPPLEMENTARY MATERIALS

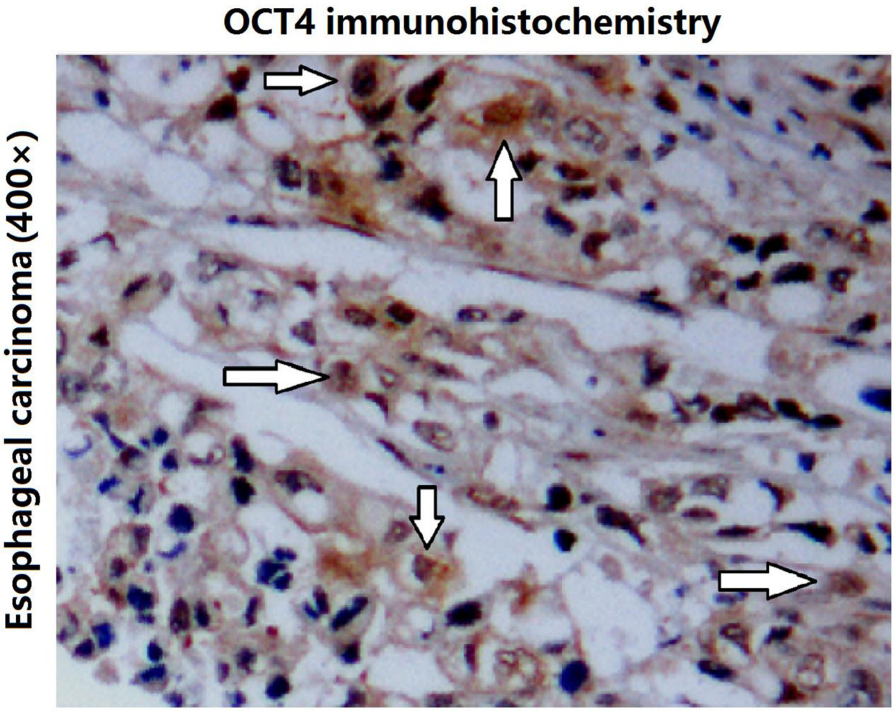

Supplementary Figure 1: OCT4 was positive mainly in nuclei of the TE1 cell xenograft tumor cells. The TE1 xenograft tumors were removed from mice and fixed in 10% formalin, paraffin-embedded, and sectioned for detecting the expression level of OCT4 by immunohistochemistry. Sections were observed under a microscope within five high-power fields (objective lens 20×) to count the percentages of positive cells. The positive OCT4 was localized mainly in nuclei (as the blank arrows showed); primary magnification: 400×.

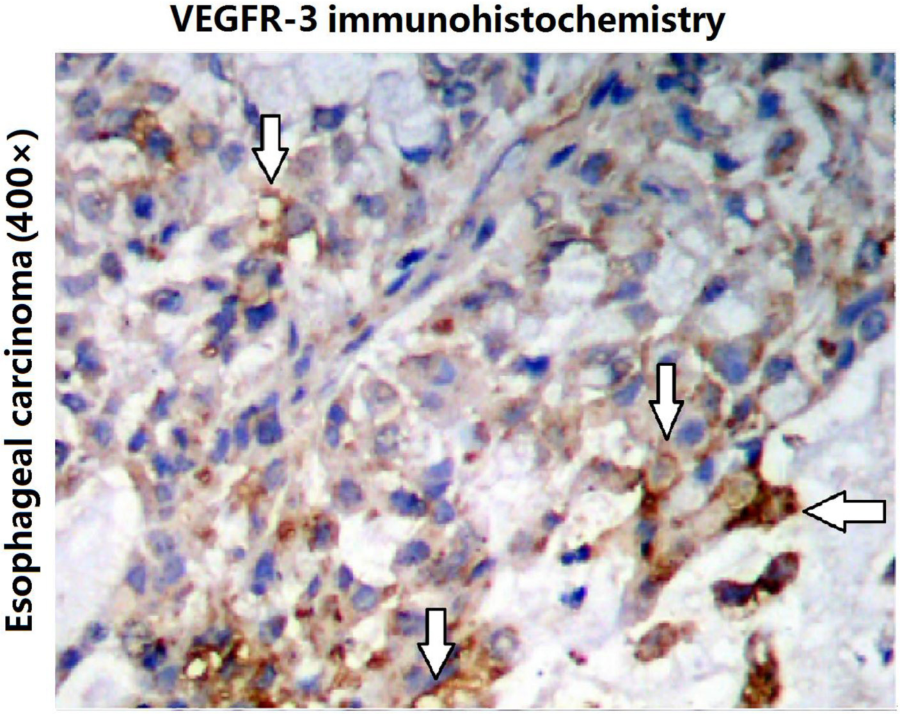

Supplementary Figure 2: VEGFR-3 was positive mainly in membrane of the TE1 cell xenograft tumor cells. The TE1 xenograft tumors were removed from mice and fixed in 10% formalin, paraffin-embedded, and sectioned for detecting the expression level of VEGFR-3 by immunohistochemistry. Sections were observed under a microscope within five high-power fields (objective lens 20×) to count the percentages of positive cells. The positive VEGFR-3 was localized mainly in membrane but it is unevenly distributed and showed granular (as the blank arrows showed); primary magnification: 400×.

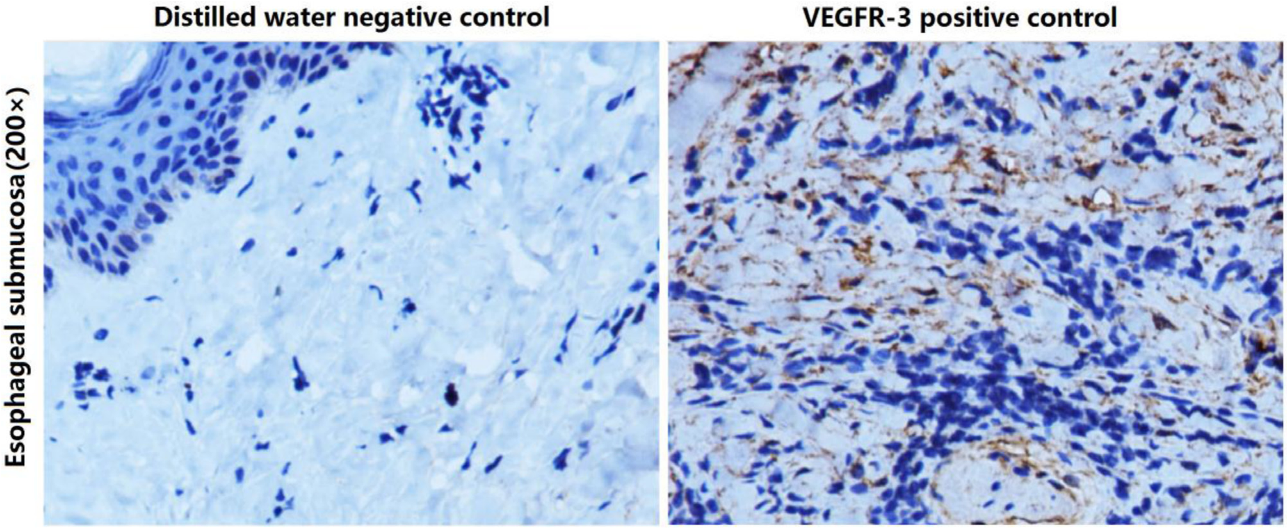

Supplementary Figure 3: Installation of immunohistochemical controls. It is generally believed that VEGFR-3 is primarily expressed in lymphatic vessels, therefore, we chose the tissue of esophageal submucosa as controls, because there are more lymphatic vessels within esophageal submucosa. The diluted water instead of VEGFR-3 antibody was set up as a negative control, and the VEGFR-3 antibody IHC staining as a positive control to show the positive expression of VEGFR-3 in lymphatic vessels.

Supplementary Table 1: Correlation between survival of ECC patients and OCT4 expression

Group		Time (m)	Status	Cumulative proportion of the survival		Cumulative number	Remaining case number
				Estimation	Standard error		
OCT4-Positive	1	12.670	Death	.933	.064	1	14
	2	13.370	Survival	.	.	1	13
	3	14.430	Survival	.	.	1	12
	4	19.200	Survival	.	.	1	11
	5	19.770	Death	.848	.100	2	10
	6	21.330	Survival	.	.	2	9
	7	23.170	Death	.754	.126	3	8
	8	39.730	Survival	.	.	3	7
	9	44.470	Survival	.	.	3	6
	10	56.730	Death	.629	.155	4	5
	11	63.630	Death	.503	.168	5	4
	12	77.570	Death	.377	.166	6	3
	13	86.200	Death	.251	.151	7	2
	14	90.630	Death	.126	.117	8	1
	15	100.300	Death	.000	.000	9	0
OCT4-Negative	1	9.800	Death	.977	.023	1	42
	2	14.030	Survival	.	.	1	41
	3	19.570	Survival	.	.	1	40
	4	26.070	Survival	.	.	1	39
	5	29.000	Death	.952	.033	2	38
	6	29.570	Death	.927	.041	3	37
	7	31.900	Death	.902	.047	4	36
	8	32.730	Death	.877	.052	5	35
	9	33.370	Death	.852	.056	6	34
	10	38.870	Survival	.	.	6	33
	11	39.570	Death	.826	.060	7	32
	12	42.100	Death	.800	.063	8	31
	13	42.100	Survival	.	.	8	30
	14	43.070	Death	.773	.067	9	29
	15	49.800	Death	.747	.069	10	28
	16	51.830	Survival	.	.	10	27
	17	52.300	Survival	.	.	10	26
	18	56.000	Survival	.	.	10	25
	19	58.900	Death	.717	.073	11	24
	20	64.530	Survival	.	.	11	23
	21	68.170	Survival	.	.	11	22
	22	71.530	Survival	.	.	11	21
	23	73.070	Survival	.	.	11	20
	24	74.330	Death	.681	.078	12	19
	25	78.000	Survival	.	.	12	18
	26	81.370	Death	.643	.082	13	17
	27	83.630	Death	.605	.085	14	16
	28	84.330	Death	.567	.088	15	15
	29	86.200	Death	.530	.090	16	14
	30	86.200	Survival	.	.	16	13
	31	90.630	Survival	.	.	16	12
	32	91.670	Death	.485	.093	17	11
	33	93.070	Survival	.	.	17	10
	34	94.000	Death	.437	.095	18	9
	35	94.530	Survival	.	.	18	8
	36	95.830	Death	.382	.098	19	7
	37	97.000	Survival	.	.	19	6
	38	100.300	Survival	.	.	19	5
	39	103.000	Death	.306	.104	20	4
	40	104.330	Death	.229	.102	21	3
	41	104.530	Survival	.	.	21	2
	42	106.070	Death	.	.	22	1
	43	106.070	Death	.000	.000	23	0

Supplementary Table 2: Correlation between survival of ECC patients and VEGF-C expression

Group		Time (m)	Status	Cumulative proportion of the survival		Cumulative number	Remaining case number
				Estimation	Standard error		
VEGFC-Positive	1	9.800	Death	.964	.035	1	27
	2	14.030	Survival	.	.	1	26
	3	19.570	Survival	.	.	1	25
	4	21.330	Survival	.	.	1	24
	5	23.170	Death	.924	.052	2	23
	6	29.000	Death	.884	.063	3	22
	7	29.570	Death	.844	.072	4	21
	8	31.900	Death	.804	.079	5	20
	9	32.730	Death	.763	.085	6	19
	10	33.370	Death	.723	.089	7	18
	11	38.870	Survival	.	.	7	17
	12	39.730	Survival	.	.	7	16
	13	43.070	Death	.678	.094	8	15
	14	44.470	Survival	.	.	8	14
	15	56.730	Death	.630	.099	9	13
	16	64.530	Survival	.	.	9	12
	17	68.170	Survival	.	.	9	11
	18	71.530	Survival	.	.	9	10
	19	77.570	Death	.567	.107	10	9
	20	78.000	Survival	.	.	10	8
	21	84.330	Death	.496	.115	11	7
	22	86.200	Death	.	.	12	6
	23	86.200	Death	.354	.118	13	5
	24	90.630	Death	.283	.114	14	4
	25	93.070	Survival	.	.	14	3
	26	94.000	Death	.189	.108	15	2
	27	95.830	Death	.094	.086	16	1
	28	100.300	Death	.000	.000	17	0
VEGFC-Negative	1	12.670	Death	.967	.033	1	29
	2	13.370	Survival	.	.	1	28
	3	14.430	Survival	.	.	1	27
	4	19.200	Survival	.	.	1	26
	5	19.770	Death	.929	.048	2	25
	6	26.070	Survival	.	.	2	24
	7	39.570	Death	.891	.060	3	23
	8	42.100	Death	.852	.069	4	22
	9	42.100	Survival	.	.	4	21
	10	49.800	Death	.811	.076	5	20
	11	51.830	Survival	.	.	5	19
	12	52.300	Survival	.	.	5	18
	13	56.000	Survival	.	.	5	17
	14	58.900	Death	.764	.086	6	16
	15	63.630	Death	.716	.093	7	15
	16	73.070	Survival	.	.	7	14
	17	74.330	Death	.665	.099	8	13
	18	81.370	Death	.614	.104	9	12
	19	83.630	Death	.563	.107	10	11
	20	86.200	Survival	.	.	10	10
	21	90.630	Survival	.	.	10	9
	22	91.670	Death	.500	.112	11	8
	23	94.530	Survival	.	.	11	7
	24	97.000	Survival	.	.	11	6
	25	100.300	Survival	.	.	11	5
	26	103.000	Death	.400	.127	12	4
	27	104.330	Death	.300	.128	13	3
	28	104.530	Survival	.	.	13	2
	29	106.070	Death	.	.	14	1
	30	106.070	Death	.000	.000	15	0

Supplementary Table 3: Correlation between survival of ECC patients and both OCT4 and VEGF-C expression

Group		Time (m)	Status	Cumulative proportion of the survival		Cumulative number	Remaining case number
				Estimation	Standard error		
OCT4+VEGFC+	1	9.800	Death	.909	.087	1	10
	2	14.030	Survival	.	.	1	9
	3	19.570	Survival	.	.	1	8
	4	23.170	Death	.795	.131	2	7
	5	29.000	Death	.682	.154	3	6
	6	29.570	Death	.568	.165	4	5
	7	31.900	Death	.455	.166	5	4
	8	32.730	Death	.341	.159	6	3
	9	44.470	Survival	.	.	6	2
	10	56.730	Death	.170	.144	7	1
	11	100.300	Death	.000	.000	8	0
OCT4+	1	19.770	Death	.750	.217	1	3
	2	42.100	Death	.500	.250	2	2
	3	71.530	Survival	.	.	2	1
	4	86.200	Death	.000	.000	3	0
VEGFC+	1	33.370	Death	.929	.069	1	13
	2	38.870	Survival	.	.	1	12
	3	39.730	Survival	.	.	1	11
	4	43.070	Death	.844	.102	2	10
	5	56.000	Survival	.	.	2	9
	6	64.530	Survival	.	.	2	8
	7	68.170	Survival	.	.	2	7
	8	77.570	Death	.724	.142	3	6
	9	78.000	Survival	.	.	3	5
	10	84.330	Death	.579	.172	4	4
	11	86.200	Death	.434	.180	5	3
	12	90.630	Death	.289	.168	6	2
	13	93.070	Survival	.	.	6	1
	14	95.830	Death	.000	.000	7	0
OCT4-VEGFC-	1	12.670	Death	.962	.038	1	25
	2	13.370	Survival	.	.	1	24
	3	26.070	Survival	.	.	1	23
	4	39.570	Death	.920	.055	2	22
	5	42.100	Survival	.	.	2	21
	6	49.800	Death	.876	.067	3	20
	7	51.830	Survival	.	.	3	19
	8	52.300	Survival	.	.	3	18
	9	58.900	Death	.827	.079	4	17
	10	63.630	Death	.779	.088	5	16
	11	73.070	Survival	.	.	5	15
	12	74.330	Death	.727	.096	6	14
	13	81.370	Death	.675	.103	7	13
	14	83.630	Death	.623	.107	8	12
	15	86.200	Survival	.	.	8	11
	16	90.630	Survival	.	.	8	10
	17	91.670	Death	.561	.113	9	9
	18	94.000	Death	.498	.116	10	8
	19	94.530	Survival	.	.	10	7
	20	97.000	Survival	.	.	10	6
	21	100.300	Survival	.	.	10	5
	22	103.000	Death	.399	.129	11	4
	23	104.330	Death	.299	.130	12	3
	24	104.530	Survival	.	.	12	2
	25	106.070	Death	.	.	13	1
	26	106.070	Death	.000	.000	14	0
